# “Why would I need more?”: How age shapes UK consumers’ perceptions of dietary protein and responses to educational interventions

**DOI:** 10.3389/fnut.2026.1893639

**Published:** 2026-07-17

**Authors:** Holly Giles, Charlotte Clark, Lisa Methven, Stephanie P. Bull, Victoria Norton, Joe Gallagher, Marianthi Faka, Elisa Glover, Stella Lignou

**Affiliations:** 1 Department of Food and Nutritional Sciences, Sensory Science Centre, University of Reading, Reading, United Kingdom; 2 Institute of Biological, Environmental & Rural Sciences, Aberystwyth University, Aberystwyth, Ceredigion, United Kingdom; 3 Arla Foods Ingredients, Viby, Denmark

**Keywords:** barriers, benefits, dietary protein, education, knowledge, population health

## Abstract

Adequate protein intake has an important role in maintaining musculoskeletal health, independence and strength during ageing. To design age-appropriate nutritional interventions, it is necessary to understand public knowledge, attitudes and perceptions surrounding dietary protein. Thus, the study aimed to investigate knowledge of protein requirements, functions, and sources, as well as attitudes towards educational materials surrounding these topics. This was addressed through an online survey with 341 consumers (74% female, 66% residing in Southeast England): here it was highlighted that participants aged 18–89 years had poor knowledge regarding protein functions, a high meat-dependency for protein sources, and a desire to learn about more plant-based protein sources. Notably only 25.8% of participants correctly identified the recommended protein intake, highlighting low levels of population knowledge. This was further explored through focus groups with younger and older adults (*n* = 15 aged 18–30, *n* = 17 aged 65+), where a knowledge deficit was seen independent of age. The most common barriers to high protein consumption were confusion, misinformation, cost, and motivation. Whilst younger adults expressed a preference for supplementation, older adults were sceptical about the need for these products. Awareness regarding the benefits of protein was low in all participants, highlighting this as a potential avenue to increase motivation for consumption. Older adults expressed a preference for educational interventions in a paper format, whereas younger adults preferred digital content and videos. There was a unanimous desire for more trustworthy nutritional information. These findings provide insight that will facilitate the development of better educational materials to increase the nutritional knowledge of UK consumers.

## 1 Introduction

### 1.1 Habitual protein consumption by the UK population

Protein is an essential macronutrient with functions including growth, muscle strength, cognitive development, cardiac health, and regulation of the hormonal and immune systems (1). Current UK guidelines recommend a reference nutrient intake (RNI) of 0.75 g of protein per kilogram of body weight per day, irrespective of age and gender. Increasing evidence suggests a higher level may have additional benefits for muscle protein synthesis in older adults (2, 3), leading to the British Dietetic Association’s recommendation of 1–1.2 g/kg/day for this demographic (4). This is in line with the European Society for Clinical Nutrition and Metabolism (2). Despite this benefit, Morris et al. (3) found that 47.5% of older males and 25% of older females are failing to meet this RNI for protein intake. This is of clinical significance, as low protein intake compounds common physical age-associated complications including fracture risk, frailty, sarcopenia, cognitive decline and overall quality of life (5, 6). This is due to the important functions of protein on muscle, digestion, immunity and tissue regulation. Demonstrating this whole body benefit of protein for healthy ageing is the finding of a positive association between higher protein intake and reduced fracture risk (7).

It is recognised that consuming protein at a safe level is essential as whilst insufficient protein intake is associated with negative health outcomes, the same is also true of excessive protein intake which can be detrimental for renal function (8). It is known that the majority of protein is commonly consumed in one meal (9): distributing protein intake evenly across the day has additional benefits on muscle protein synthesis (3, 10–12). Thus, identifying methods for increasing protein intake at breakfast, lunch and between meals presents a promising nutritional strategy for increasing total daily protein intake (6, 13). Considering the ageing population of the UK (14), it is essential that protein needs are met across all demographics to improve health outcomes at a population level and protect older adults from the potential health consequences of malnutrition (50).

### 1.2 Barriers to increasing protein consumption

In keeping with improving population health, there is a need to understand the protein sources commonly chosen. Proteins in foods and beverages can originate from both animal (meat, eggs, dairy) or plant (legumes, pulses, nuts, grains and seeds) sources and it is the position of the Academy of Nutrition and Dietetics that vegetarian and vegan dietary patterns can be nutritionally adequate and offer long-term health benefits (15, 49). It is of note that plant-based protein sources are likely to have higher fibre content, providing additional health benefits (16). Despite this, in the UK, animal-based foods are the dominant dietary protein source (17) and research shows that attempts at increasing protein intake usually result in an increase in meat consumption (18). A study with Dutch older adults, reported a need for enhanced knowledge and tools about the health and environmental benefits of plant-based, protein-rich diets to enable adoption of this dietary pattern (19). Barriers to plant-based protein consumption have been listed as: a belief that humans are meant to eat a lot of meat; the expectation of a poor sensory profile for plant-based products; and the belief that plant sources of protein would not provide sufficient energy or strength (20). This work was completed in 10 countries across Europe meaning the specific barriers for UK consumers are not fully understood. A more thorough understanding of the attitudes of consumers of all ages towards protein sources and requirements is anticipated to facilitate more accurate interventions, leading to higher engagement and more sustainable dietary change.

The barriers to increasing protein intake by older adults were summarised by Smith et al. (6), to include poor appetite, a high satiating effect of protein, food neophobia, dexterity issues impacting food preparation, limited oral manipulation abilities and the increased chewing requirements of protein-rich foods, and a decline in taste and aroma perception which impacts enjoyment. Geny et al. (21) showed that home cooking following protein-fortified recipes can reduce older adult’s barriers to using high-protein ingredients. This home cooking approach was also reported in Ireland with older adults choosing to prepare their own high protein foods, as opposed to buying the products in the market (22). This finding highlights knowledge and confidence as barriers to high protein consumption for this demographic (21). This was seconded following dietary counselling with Dutch consumers, which successfully increased protein intake and sustainable dietary change by older adults as a result of increased knowledge (18). Existing research has largely focused on older adults, meaning the barriers preventing varied protein consumption by younger adults is not fully understood.

Barriers to increased protein consumption assume that there is a desire to increase protein intake. Smith et al. (23) previously highlighted a lack of awareness among older adults regarding their increased need for protein during the ageing process. This was seconded by a focus group study in older adults which reported that participants were unaware of the potential for dietary protein inadequacies (24). It has also been reported that older adults cite confusion, distrust and personal relevance as barriers to accepting protein-enriched foods (25), which may be reflective of a lack of desire to increase intake. It is not known if this age-group specific or indicative of a larger inadequacy in nutritional knowledge across the population. Thus, there is a need for research with both younger and older adults to understand the facilitators and barriers to adequate protein consumption.

### 1.3 Sources of nutritional information to improve eating behaviours

Educational materials have been shown to increase protein intake in patient groups: Ota et al. (26) reported that education during pregnancy to increase protein intake is an effective measure in reducing the risk of preterm births and low birth weights (26). Whilst this highlights the ability of educational interventions to modify behaviour, the reliance on leaflets, posters and face-to-face sessions may not be the most appropriate or engaging tools for inciting behavioural change. The use of digital sources, including social media, is growing across all demographics, meaning that research is needed into educational interventions via this medium. 64.8% of all internet users have been shown to use the internet to search for information on diet, nutrition, vitamins and nutritional supplements (27); these figures are expected to have further increased to the current day due to the global rise in social media use and digital content consumption. Previous research showed that preferences for sources of nutrition information are determined by age, social relationships, and electronic competencies (27), with younger adults expressing a greater interest in using the internet to access nutrition information. It is possible that this may indicate the need for age-specific interventions to increase engagement with the target consumer, however, the authors recognise the high levels of heterogeneity present within older adults regarding technology use meaning this approach should be considered for all. Increased understanding of the use of digital information sources, and age-specific preferences, is needed to facilitate accurate and relevant information design.

Population health is also guided by figures of trust such as doctors, chefs, or celebrities. This landscape is changing through the growth of social media influencers (SMI) giving more people access to share their views with a mass audience: these may pose an important avenue for promoting behavioural change, especially with younger adults. SMI with 10,000–100,000 followers, considered micro-influencers, have been identified as effective at promoting dietary changes using a stereotypically attractive individual that appears connected to the audience with styles of content delivery to generate a high level of audience trust (28). It has been suggested that the methods used by SMI could be utilised by nutritionists to build trust in educational messages about food and nutrition. This justifies the need for further research into educational interventions and the use of technology within this space to engage with participants of all ages to encourage positive behavioural change regarding protein intake.

### 1.4 Aims and objectives

There is a need to understand population knowledge and attitudes towards protein intake, with particular focus on the barriers and facilitators to adequate protein consumption. The use of traditional educational interventions has been discussed; it is not known if this reflects the preferences of participants to increase knowledge and trust in the food system. Thus, the potential for technology including social media should be investigated to enable more engaging and relevant educational interventions for younger and older adults to increase sustainable protein intake from a range of sources. In light of this, the present study aimed to: (1) investigate participants’ knowledge of protein requirements, functions and sources; (2) explore participants’ attitudes towards protein fortification and supplementation; (3) understand participants’ age-associated attitudes towards educational materials; and (4) gain feedback on the best formats for educational materials including social media with different demographics. It is hoped that this increased knowledge would facilitate the development of more relevant educational materials, leading to increased engagement and healthier protein intake in the UK. A survey was used to understand the level of knowledge surrounding dietary protein across the UK. It also addressed attitudes towards fortification and supplementation. This was investigated further using focus groups to understand participant motivations, barriers, and perceptions of high-protein foods. Focus groups also tackled the effect of interventions using educational messaging and compared the effect of age on educational preferences.

## 2 Methodology

### 2.1 Study outline

The study consisted of two parts: a survey with UK adults of any age; and focus group sessions with two targeted groups (young adults and older adults). For the first part of the study, 341 participants aged 18–89 years (41.4 ± 17.3) completed an online survey between December 2023 to January 2024. The sample size was calculated in accordance with Yamane’s formula ( $n=\frac{N}{1+Ne^{2}}$ , where *n* = sample size; *N* = population, and *e* = precision of 0.06), which indicated that a minimum of 277 participants was sufficient (53). Participants were recruited from across the UK via the University of Reading volunteer databases (Hugh Sinclair Nutrition database and Sensory Science Centre database), various social media platforms and university departmental email lists. Before completing the survey, participants provided informed consent, were informed that their response would be anonymous and that they could withdraw from the study at any point. To maximise the representation of the survey across the UK, the only exclusion criteria was participants aged under 18 or living outside the UK at the time of distribution.

For the second part, 32 adults were recruited (*n* = 15 aged 18–30, *n* = 17 aged 65+) to take part in focus groups at the Sensory Science Centre at the University of Reading during July 2024. It was estimated that two sessions (with an average of 8 consumers per focus group) for each age group would be sufficient to cover more than 80% of all themes (29). The effect of gender, ethnicity or socio-economic factor was outside the scope of the present study, meaning the relatively small sample size was believed to be sufficient to conclude on age-associated differences. Inclusion criteria for the focus groups were: participants aged 18–30 or 65 and over, living in the UK, able to attend the university for an in-person session, with no self-reported prior formal nutritional education. Similarly to the online survey, consumers had the study explained, provided informed consent, and were notified that the data would be pseudo-anonymised and that they were able to withdraw at any time.

The study was conducted in accordance with the Declaration of Helsinki and was given a favourable opinion for conduct by the School of Chemistry, Food and Pharmacy Research Ethics Committee at the University of Reading (study number: 66/2023).

### 2.2 Survey

#### 2.2.1 Design

This aspect of the project aimed to gather insights into participant’s knowledge, awareness, attitudes and behaviours related to dietary protein, as well as their preferred way of receiving information that could support the development of future educational resources. The survey was deployed online via the Compusense platform (Version 21.0.7713.26683, Compusense, Canada). Participants were initially presented with a short introduction about the project followed by an informed consent from. The survey was organised into five parts and included several questions formats such as single-choice items (e.g., yes/no/unsure), check-all-that-apply (CATA), ranking and open-ended questions: the questions and possible responses are detailed in Supplementary Table S1. The first part aimed to understand participants’ awareness, knowledge and perception of dietary protein in terms of content in different foods, recommended intake and functions in the body, awareness of protein requirements changing with age and the population group most at risk of protein deficiency. The second part focused on knowledge and perception of protein supplements and fortified foods followed by consumption habits and motivations of the latter. Participants were also questioned on the barriers preventing them from consuming fortified foods and whether they thought there was any harm in taking protein supplements. The third part explored the health benefits of consuming protein and the factors that would motivate participants to consume more protein, followed by the fourth part that investigated participants’ willingness to learn more about dietary protein, the most trusted sources of information, and their preferred formats. The final part outlined basic demographic information including age, gender, ethnicity, living location, diet style, educational level and yearly income.

#### 2.2.2 Statistical analysis

Cochran’s *Q* test was used for the check-all-that-apply questions using McNemar’s test and Bonferroni adjustment for multiple pairwise comparisons (51). Friedman’s test was deployed on ranked data with Nemenyi *post hoc* test for pairwise comparisons. For both tests a significance level of *p* < 0.05 was chosen to assess significance between responses. Frequency of responses falling under recurring themes was used for open-ended questions (52). All analyses were conducted using XLSTAT (version 2025.1.1.1429, Addinsoft, USA).

### 2.3 Focus group sessions

#### 2.3.1 Design

Results from the survey were subsequently used to inform the development of a semi-structured discussion guide used in the focus group sessions. This was centred around five key themes: protein requirements, functions, sources, attitudes towards fortification/ supplementation, and educational interventions (Table 1). All sessions were conducted by the same facilitator in July 2024 to increase consistency. Sessions were audio recorded using Microsoft Teams (version 26015.1706.4351.4392, Washington, USA) and auto-transcribed. A second moderator was present for all focus groups to write notes of key discussion themes which were discussed among the research team immediately after completion of the focus groups: these discussions formed the basis for the codes used for analysis.

**Table 1:** Semi-structured discussion guide used for the focus groups including the five key themes and the planned questions that were used as conversation prompts during the sessions.

Theme	Questions
Protein requirements	Do you think there is a particular demographic that needs more protein?
Protein functions	What do you think the body needs protein for/ what is the function of protein in the body?
Protein sources	Do you believe you eat enough protein, and what foods are you eating to meet this?
	From your perspective are there any barriers to consuming more protein?
	Do you think that a particular type of protein is better than any other?
Protein fortification/supplementation	Have you heard of fortified food, specifically fortified foods with protein? And if so, do you eat any?
	Have you heard of protein supplements? And if so, do you take any?
	Do you know what the difference between supplements and fortified food is?
Educational interventions	Do you trust this information given to you?
	Is there anything from these leaflets that was news to you/is there anything that you learnt from this?
	Which of the two did you prefer learning from and if you were to receive any more information what would your preferred format be?
	Do you think if people were more informed by leaflets such as these on the benefits and functions of protein on the body, they would change their diet?
	Now you have read this information do you believe you eat enough protein, has your opinion changed on your own diet?

At the start of the session consumers were informed how the session would work (2 × 45-min discussions with a 15-min comfort break), and an emphasis was made for all participants to contribute verbally and that any answers were acceptable and desired. All focus groups started with an icebreaker task asking participants to share if they think they eat enough protein and what foods are most likely to contribute to their protein intake. The first half of the session focused on the participants’ knowledge of protein sources, functions and requirements. This led into the barriers to consuming more protein, and their attitudes towards high-protein foods such as fortified products and supplements. Supplementary questions were used to expand on answers at the moderator’s discretion depending on the talkative nature of the group.

**Figure 1:**
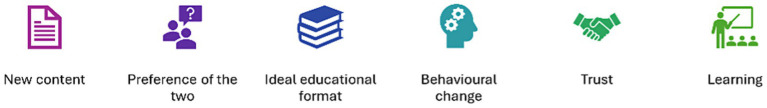
Summary of the themes of key questions used in the focus groups to evaluate the educational materials based on resources from the British Nutrition Foundation and European Food Information Council.

During the break participants were given two educational leaflets, which had been adapted from the British Nutrition Foundation (Supplementary Figure S1) and European Food Information Council (EUFIC) (Supplementary Figure S2). The first leaflet was more text-based, and the second one was an infographic. Participants were given 10 min to read both sources and then they answered questions surrounding their preferences between the two leaflets, what they learnt, what they liked about them, and what factors they would use in determining whether to trust the information provided (Figure 1). This led into wider discussions about the format of educational materials, including the use of technology and social media to increase awareness of the topics discussed. Finally, participants were asked whether the topics discussed in the session were likely to lead to a change in their protein consumption in the coming weeks. After this, participants were given the chance to ask any questions to the facilitator and moderator.

#### 2.3.2 Focus group analysis

The automatic transcription created using Microsoft Teams was manually edited using the video recording of the session by researcher CC. The transcription was then independently checked by two other researchers from the study team (HG and SL). The transcribed data was coded in NVivo (release 15.3.1, Denver, USA) to identify, analyse, and report emerging themes using an inductive data-driven approach. Initial codes were generated by the research team through discussions immediately after the sessions, themes were developed, reviewed and defined during the analysis process (30). The coding was completed in a short timeframe to reduce the subjectivity that is inherent to this method. This was minimised further by the codebook being cross-checked by two other researchers to ensure accurate data representation was being achieved. Quotes have been presented followed by the gender and age of the participant to provide context whilst maintaining anonymity, for example (M, 78) refers to a 78-year-old male.

## 3 Results

### 3.1 Survey

#### 3.1.1 Demographics

A total of 341 participants completed the survey as shown in Table 2. The cohort were mainly female (74.8%), following an omnivore diet (73.6%), living in Southeast England (66.3%), having an undergraduate degree (39.3%), with no nutrition-related degree (84.2%) and with the highest proportion having an individual yearly income between £12,571–50,270 (49.6%).

**Table 2:** Survey participants’ (*n* = 341) demographic overview.

Category	*n*	%
Gender		
Male	83	24.3
Female	255	74.8
Prefer not to say	3	0.9
Living location		
South-east England	226	66.3
South-west England	54	15.8
Midlands	23	6.7
North-east England	12	3.5
North-west England	11	3.2
Wales	10	2.9
Scotland	1	0.3
Northern Ireland	4	1.2
Education level		
GCSE	26	7.6
A levels	59	17.3
Undergraduate degree	134	39.3
Postgraduate degree	100	29.3
PhD	19	5.6
None	3	0.9
Nutrition-related degree		
Yes	47	13.8
No	287	84.2
Unsure	7	2.1
Diet style		
Omnivore	251	73.6
Pescatarian	10	2.9
Vegetarian	27	7.9
Vegan	9	2.6
Flexitarian	37	10.9
Religious related	1	0.3
Other	6	1.8
Ethnicity		
Indian	8	2.3
Arab	2	0.6
Chinese	3	0.9
Black	11	3.2
White and black African	2	0.6
White and Asian	3	0.9
White	301	88.3
Other	11	3.2
Yearly income		
Up to £12,570	35	10.3
Between £12,571 and £50,270	169	49.6
Between £50,271 and £125,140	48	14.1
Over £125,240	10	2.9
Prefer not to say	79	23.2

#### 3.1.2 Knowledge of protein sources and recommended intake

**Figure 2:**
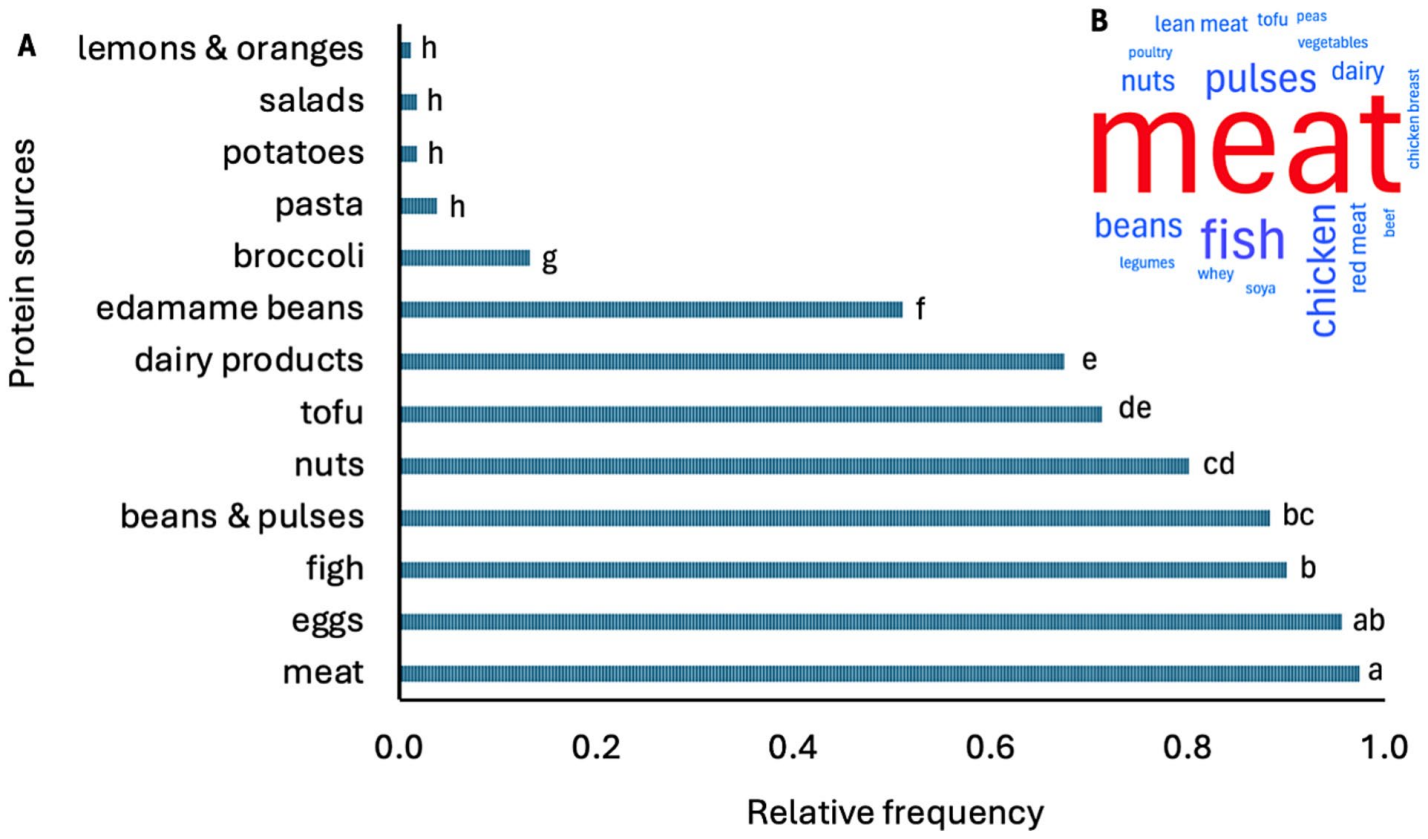
Participants’ responses (*n* = 341) on good sources of proteins: **(A)** food items (data are reported as relative frequencies and differing letters indicate statistically significant differences based on Bonferroni multiple comparison test with a significance value of 0.05); **(B)** key words identified in open-ended question: “What do you think the best source of protein is?”.

Participants chose meat, eggs, fish, dairy products, beans and pulses, nuts, tofu, and edamame beans as good sources of protein significantly more often than pasta, salads, lemons and oranges (*p* < 0.0001; Figure 2A) highlighting some knowledge of high protein foods. When asked which is the best protein source, the majority indicated meat, fish or eggs (Figure 2B). Participants were asked whether they believed food sources are sufficient for their protein requirements (meaning their nutritional needs can be met without need for protein supplementation or fortification) with 68% answering yes, 10% no and 22% unsure.

Five options were provided for the daily protein recommended intake: 0.5, 0.75, 1, 1.25 g per kg of body weight per day, or “I do not know” (Supplementary Table S1) and of these options, 25.8% of participants answered correctly (0.75 g/kg/day). From the remaining participants, 36.7% did not know the recommendation, 9.4% underestimated and 28.1% overestimated the amount, respective to the RNI. In addition, 86.2% of participants answered that protein requirements change with age, whereas 9.4% were unsure. Participants that positively answered that protein requirements change with age were further questioned if they think that adults over 65 require more or less protein than their younger counterparts, with 40.8% answering more, 50.3% less and 8.8% stating that needs would remain the same suggesting they were referring to a different age-associated change (children, pregnancy, etc.). In terms of which population group is most at risk of protein deficiency, 36.1% selected those over 65, 26.4% selected children, 24.3% chose pregnant women, and 13.2% were unsure.

#### 3.1.3 Protein supplements and fortified foods

**Figure 3:**
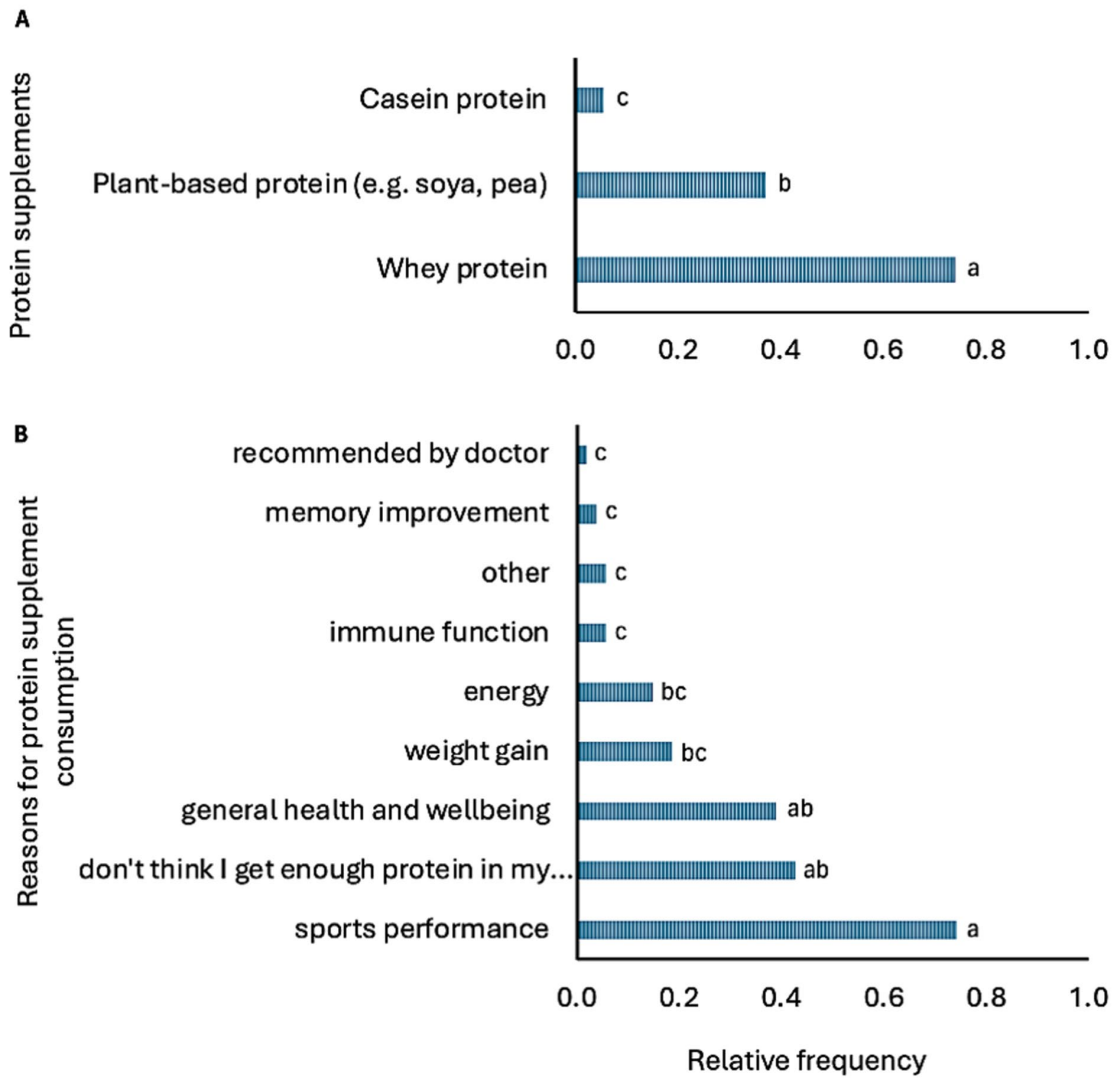
Survey participants that consume protein supplements (*n* = 54): **(A)** type of supplement; **(B)** consumption reasons (data are reported as relative frequencies and differing letters indicate statistically significant differences based on McNemar (Bonferroni) multiple comparison test).

The majority of participants (84.2%) indicated that they do not take protein supplements. Those that indicated that they regularly consume protein supplements were asked to specify the type of supplement and the reason for consuming them. Participants reported whey protein as the most common supplement (*p* < 0.0001; Figure 3A) and significant differences were observed between the reasons behind their consumption (*p* < 0.0001; Figure 3B). Sports performance was the most common reason provided for consuming protein supplements (Figure 3B). Furthermore, whilst 40.4% of participants reported that they were unsure whether there is any potential harm caused by consuming protein supplements, 33.4% did not think protein supplements were harmful, and 26.1% believed they would cause harm. Due to the quantitative nature of the survey, it was not possible to investigate the reasons for this belief, however this was further explored in the focus groups (Section 3.2).

**Figure 4:**
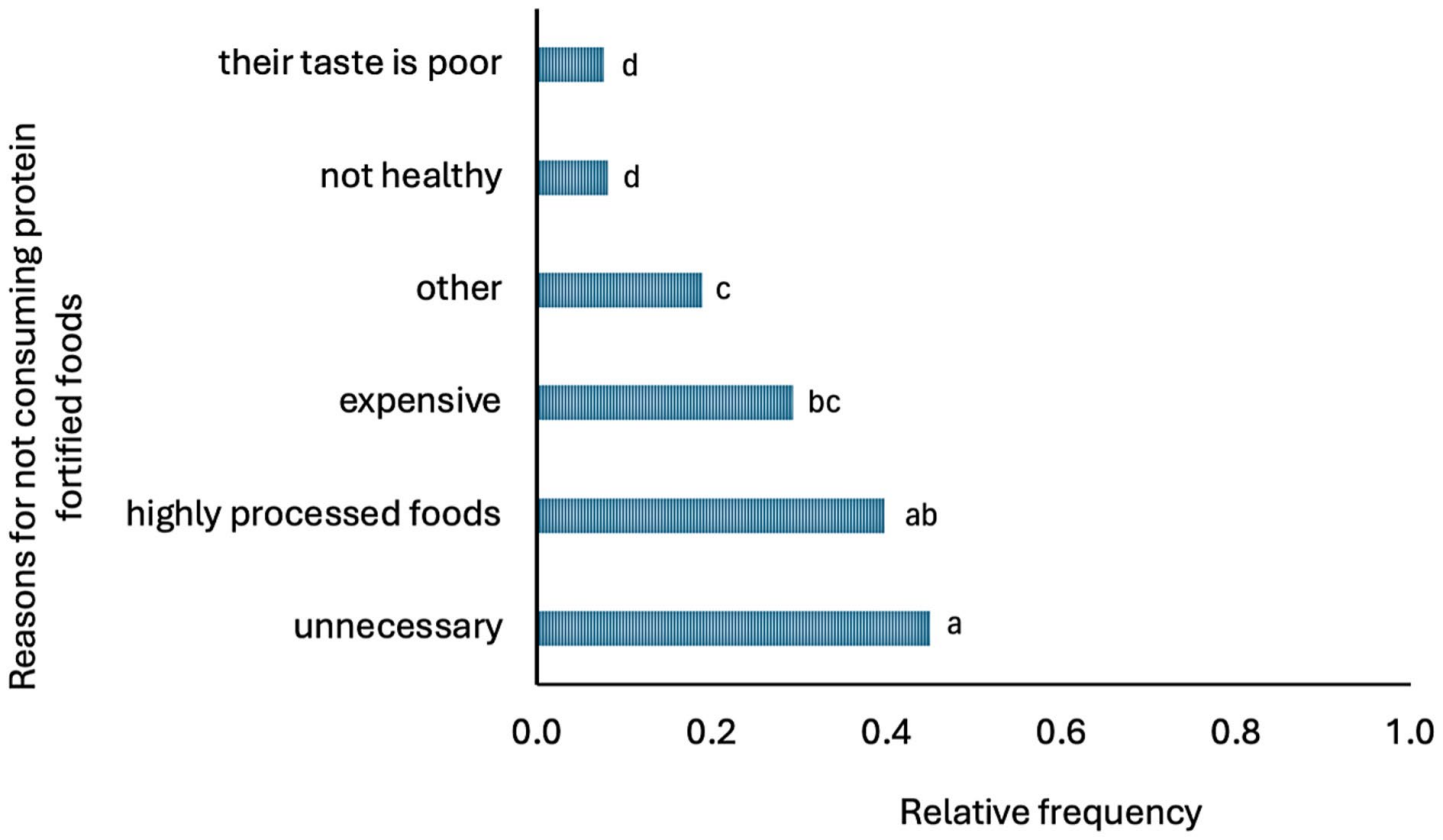
Reasons for not consuming protein fortified foods (*n* = 249; data are reported as relative frequencies and differing letters indicate statistically significant differences based on McNemar (Bonferroni) multiple comparison test).

Most participants (74.2%) knew what fortified foods are, whereas 17.3% did not know, and 8.5% were unsure. Subsequently, 65.1% reported that they do not purchase and consume fortified foods. Those that answered no or unsure to consuming these products (73%), were further asked the reasons behind this response and there was high consensus that fortified foods are unnecessary, highly processed or expensive (*p* < 0.0001; Figure 4).

#### 3.1.4 Health benefits

**Figure 5:**
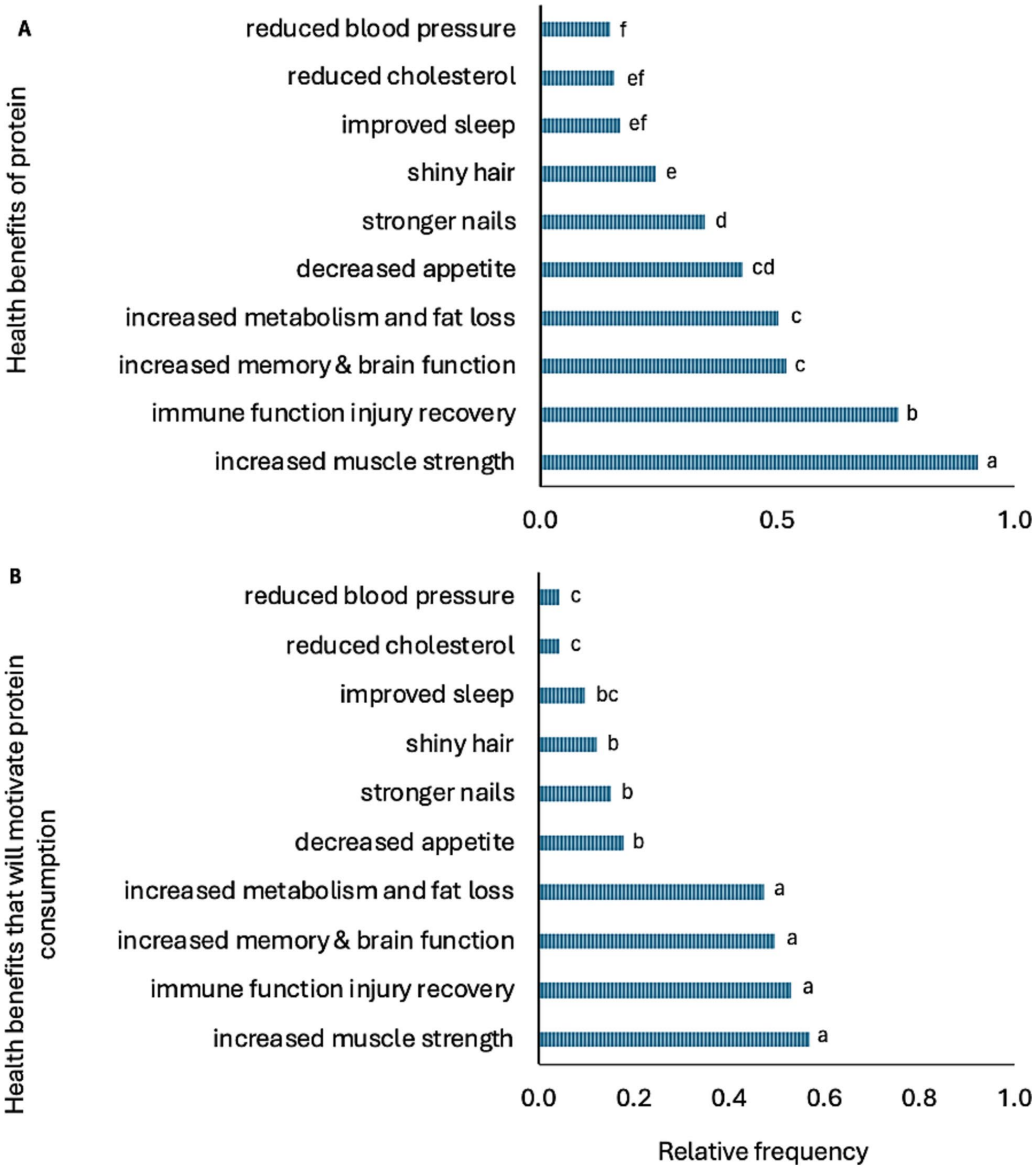
**(A)** Health benefits of protein and **(B)** Health benefits that will motivate participants to consume more protein (*n* = 341; data are reported as relative frequencies and differing letters indicate statistically significant differences based on McNemar (Bonferroni) multiple comparison test).

Participants were asked to indicate what the health benefits of protein are (Figure 5A) and which of these factors would most motivate them to consume more protein (Figure 5B). Increased muscle strength and immune function/injury recovery were the two benefits that were significantly chosen the most often (*p* < 0.0001, Figure 5A). These, as well as increased memory and brain function and increased metabolism/fat loss, would motivate participants to consume more protein (*p* < 0.0001; Figure 5B). Furthermore, the majority of the participants (72.1%) indicated that to they wanted to learn more about benefits of protein for health and where to find it in their diet.

#### 3.1.5 Type and format of information

**Figure 6:**
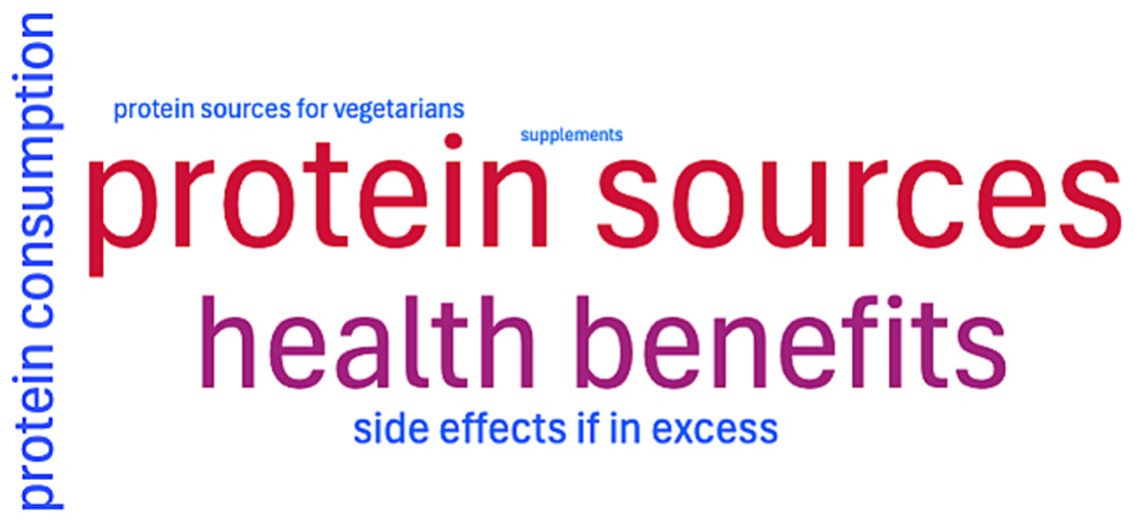
Major themes from open-ended question on what other information participants would like to know regarding protein (*n* = 341).

**Figure 7:**
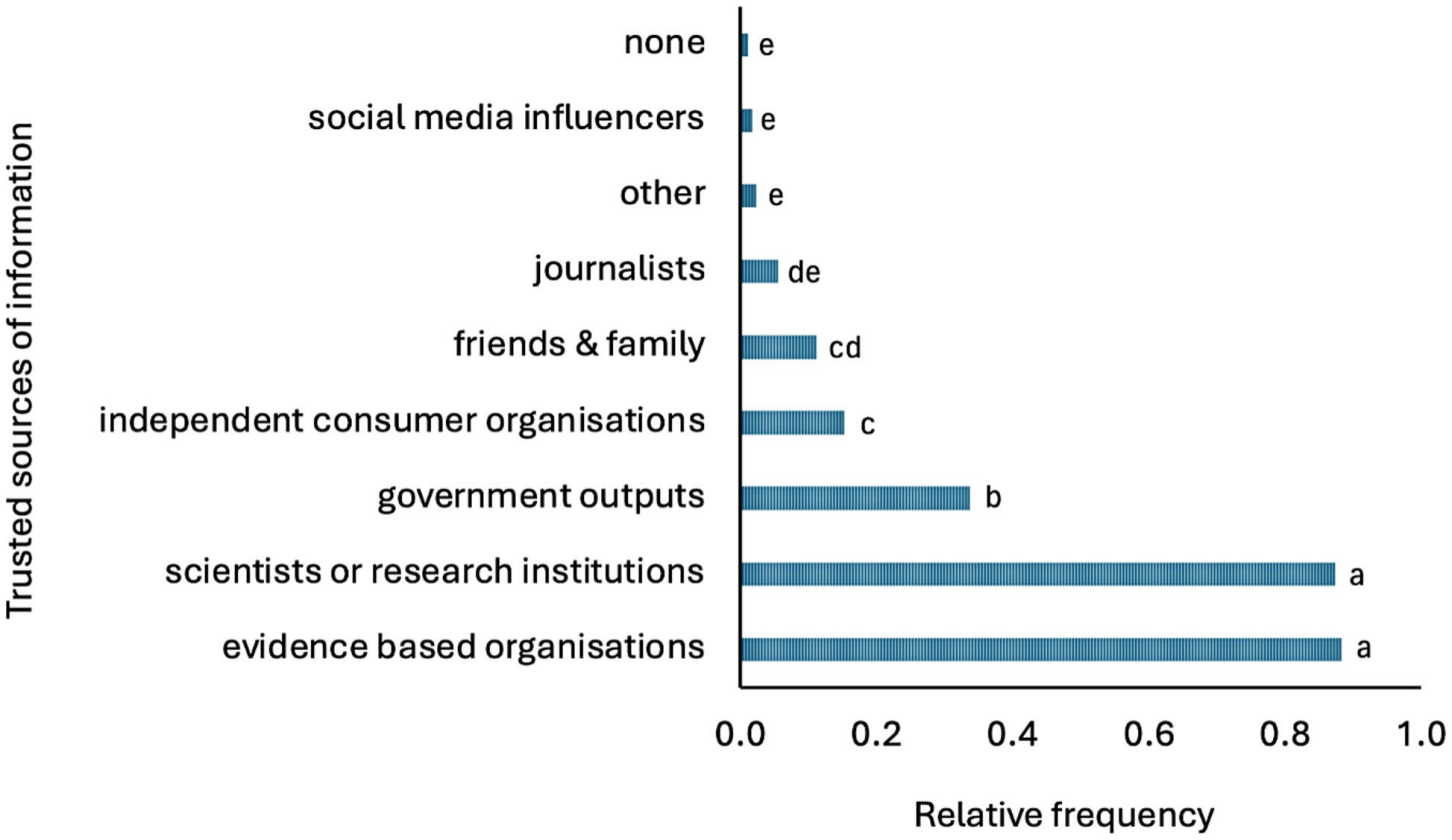
Trusted sources of information (*n* = 341; data are reported as relative frequencies and differing letters indicate statistically significant differences based on McNemar (Bonferroni) multiple comparison test).

Following the questions on health benefits of protein, participants were asked what other information they would like to know regarding protein using an open-ended question. The results demonstrated a willingness to learn more about protein sources, health benefits from consuming protein, requirements and general information (Figure 6). Subsequently, participants were asked to specify which sources of information they trust, and it was apparent that evidence-based organisations (such as the British Nutrition Foundation) as well as scientists or research institutions were the most important (*p* < 0.0001; Figure 7).

Finally, participants were requested to rank their preferred information distribution format. An online information sheet was significantly the most preferred format followed by a video to watch online (*p* < 0.0001). Face-to-face individual or group sessions were the least preferred formats overall.

### 3.2 Focus groups

#### 3.2.1 Demographics

A total of 34 participants (35.3% male, 61.8% female and 2.9% other) were recruited to take part in the 90-min focus group sessions. Of these, 19 were older adults (65–82, 71.6 ± 4.3 years) and 15 young adults (18–30, 24.8 ± 3.1 years).

#### 3.2.2 Protein requirements and functions

The majority of participants admitted that they were not sure if they were eating enough protein, or the most common protein sources (Section 3.2.3). Some younger adults were aware of the requirements as a result of using tracking apps, but general awareness of guidelines was low. Both age groups suggested that children and teenagers would need the most protein: “*children, definitely”* (F, 65) and “*if you are growing… I would just say teenagers”* (M, 69), as well as active individuals: “*it depends how active they are”* (F, 70). Limited suggestions were made about the needs of older adults. Both age groups cited muscle strength and growth as important functions of protein but were unable to name any other functions. Some older adults were aware of the satiating effect of protein: “*I understand it helps… you stay fuller for longer. So you end up ultimately eating less if you are having protein with most of the meals during the day”* (M, 68). In response to a contrasting question about the effects of protein deficiency, some potential issues were given such as lack of energy, digestive issues, poor immunity, but participants did not associate adequate protein intake as having protective effects over these systems.

#### 3.2.3 Protein sources

Both age groups associated protein with animal products: meat, fish, and dairy were given most commonly as examples of protein-rich foods. Meat sources were mentioned 26 times, whereas wholefoods were mentioned 13 times: plant-based sources of protein were only mentioned by current or previous vegetarian or vegan individuals, with the exception of meat analogues which were mentioned by young adults. This high animal-dependency and low knowledge of plant-based protein sources was identified as a barrier to increased protein consumption: with one participant describing it as “*not knowing what the other proteins are… other than meat”* (F, 24). Cost was the most cited barrier to increased protein consumption, with both age groups identifying that good quality protein is often more expensive. There was some disagreement regarding cost when discussing protein sources: supplements were identified as having a high cost: “*Supplements say like your protein shakes, the protein bars. None of it is cheap*” (F, 25); whilst beans and lentils were considered a cheaper protein source, but this was only identified by vegetarian participants. Other factors preventing varied protein consumption included boredom (due to consumers’ only being aware of a limited number of protein-rich foods), lack of awareness or need to increase intake, and confusion/ misinformation being given as common barriers (Table 3).

**Table 3:** Barriers to increased protein consumption for older and young adults including the number of references made for each barrier and some examples.

Barrier	References	Quotes
High frequency (10+ mentions)		
Cost	(16)	*Cost is an issue as well […] good quality protein…tends to be fairly expensive compared to other alternatives* (M, 68) *I was gonna say the crisis of families, if you have lots of children… I do not know how… you could not spend £14/15 on a chicken* (F, 75) *I think for some people, it’s cost* (F, 65) *I mean pulses and beans and lentils are full of protein and so cheapest* (F, 67) *Protein comes from meat and it’s expensive* (M, 67) *Vegan version of something like just chicken and rice.* Bars are so expensive as well (F, 25) *There’s very high in protein but not high everything else that’s quite expensive* (M, 30) *Meat is expensive* (F, 25) *The biggest one is like price* (NB, 21) *Supplements say like your protein shakes, the protein bars. None of it is cheap* (F, 25) *Expensive* (F, 24)
Confusion and misinformation about protein sources	(12)	*So I do find I’m getting a bit confused about you know are there sources of protein that I do not know about? Am I eating too much? […] you see so many different programmes saying you must eat this and you must need that […] quite confusing to know about what you should or should not be eating and how much […] I think I’m just in a muddle really* (F, 75) *These are mixed messages* (F, 74) *A lot of misinformation as well […] really popular on social media and they are in good physical shape, they could be misinforming people* (M, 25) *It might not even be like misinformation deliberately – this information, you know, just like survivors bias* (NB, 21)
Lack of awareness of need (no motivation)	(12)	*I do not think I need to consume any more than I do* (F, 70) *Why would I need to add anymore* (M, 67) *So I do not think we really think about whether we are getting enough proteins or carbohydrates* (F, 66) *Think the protein is the least important […] so I would probably cut out protein, but I should or not, I do not know* (M, 67) *I do think that an awful lot of the NHS is fighting fire and there should be an awful lot more of education to prevent these* (F, 67) *I have enough protein anyway – it’s about the need, I’m not aware that I need it so* (M, 67) *What am I supplementing if I’m already eating* (M, 78) *Not actually knowing exactly what I’m eating every day, yeah* (F, 25) *I just like have never been told […] I think it’s just like education* (F, 24)
Medium to low frequency (1–5 mentions)		
Ethical issues	(5)	*Aside from the obvious ethics about red meat that might stop some people eating* (M, 69) *I would just say about the ethical thing […] if we do not eat cows or whatever, they will just disappear from the landscape because farmers cannot afford to do it […] So I think if the ethical thing is a very complex thing* (F, 75)
Time	(4)	*I’m like, on the go a lot of the time it is picking stuff up which ends up not being very healthy* (F, 22) *I think it is like a ‘big commitment’* (F, 25) *I think time is a factor* (M, 25) *If I’m, like, really busy or just cannot be on the cook off, we just make pasta* (F, 28)
Cooking alone	(2)	*Sheer hassle of cooking, especially if you live by yourself […] got friends who live alone, who will get a ready meal rather than cook* (F, 70)
Boredom	(2)	*Lack of variety that you find in stores and supermarkets* (M, 78) *Like diversity, as well, like I do not want to be like having same protein shake every day or like chickpeas for every meal* (NB, 21)
Concerns about levels of processing	(2)	*…they are obsessed with eating non processed food* (F, 75) *…because it is ultra processed* (F, 73)
Unhealthy foods	(1)	*advertising for food* (F, 73)
Reduced appetite and high satiating effect	(1)	*as you get older, your appetite lessens* (F, 70)
Thermogenic response	(1)	*It increases heat in your body* (F, 24)

#### 3.2.4 Supplementation and fortification

Participants were initially asked if they were aware of protein fortified foods and protein supplements. After a discussion about their initial knowledge, a definition of the two product types was provided as well as examples such as a high-protein bagel and a protein shake, respectively. All younger adults questioned were aware of protein supplements and some reported taking them currently or previously: “*it’s quite an easy way that [you can get] your daily intake in …if you are rushing*” (M, 25) and “*that’s also why I pick a supplement… to have a little bit more control, like, I’m able to tell how much protein… like how supplemented my diet [is] without all the added extra like calories and sugar”* (F, 24). None of the younger adults regularly consumed fortified foods, but those who had previously tasted them complained about the sensory profile of fortified foods: “*I really did not like it. The protein yoghurts. it’s like super thick and not that pleasant*” (NB, 21). Older adults were all unaware of the term fortification but after explanation were familiar with the concept and displayed product familiarity for examples given such as fortified bread and cereal products. None of the participants had bought fortified products: reasons listed included concerns about additives, ultra-processing, not being healthy, and not needing additional protein. When given a choice between a protein supplement and a fortified food, younger adults cited a preference for supplements, whereas older adults would choose fortified foods.

#### 3.2.5 Educational leaflets on dietary protein consumption

During the focus group, two educational materials were provided for participants: one was more text heavy (Supplementary Figure S1) and one was more image based (Supplementary Figure S2). Both age groups preferred the infographic and found it easier to read out of the two options provided. However, when considering the wider range of possible educational interventions, younger adults expressed a preference for video formats and social media as opposed to written content. Younger adults disliked a paper format: “*if it’s paper it will sit in the house for years or go in the bin*” and “*I always feel guilty with paper”* (M, 25). However, the younger adults also expressed concerns with trust on social media and influencers: “*the internet is harder to trust, though. So… when you see like some something on the Internet, it’s almost like if it’s sponsored or something*” (M, 18). In contrast, older adults cited a preference for written information (such as the printed infographic provided) due to the ability to go back to material multiple times. It is also noted that some participants would prefer this to be a hard copy, whereas others were happy with an electronic version.

## 4 Discussion

### 4.1 Awareness of protein requirements and functions

The study indicates low knowledge of protein requirements across the population: only 25.8% of the survey participants were able to correctly identify the RNI for protein in a multiple-choice question. The high proportion of participants with higher education degrees (68.6%) means it is possible that populational awareness would be lower still. This is a limitation of the survey which the authors’ acknowledge when making interpretations. Poor awareness of RNI agrees with previous works (31) with one study reporting that 35.3% of older adults interviewed did not know was meant by the term “dietary protein” (32). It was hypothesised that younger adults would have higher levels of awareness of protein requirements than older adults; this was confirmed in the focus groups, where a higher proportion of younger adults were able to state the RNI value compared with older adults. Younger participants attributed this to the use of dietary tracking apps, highlighting the ability of these platforms to increase nutritional awareness. Whilst it is possible that these platforms present an untapped resource for older adults, it is known that concerns regarding ease of use and privacy are likely to limit uptake of nutrition tracking apps with this demographic (33). It is noted that a large proportion of those in the survey who did not know the RNI were younger adults suggesting that knowledge is low within both age groups and that nutritional tracking is either not widespread enough within this age group to correct a knowledge deficit, or that tracking alone is not sufficient. It is also possible that this reflects confusion in how to calculate the RNI for protein as this is based on body weight per person, rather than a one-size-fits-all value as for many other macronutrients. It supports the use of tracking alongside educational material to improve nutritional awareness of dietary recommendations.

In the focus groups, both age groups thought that those with more active lifestyles, including children and teenagers, would require a higher protein intake. Some participants cited *“the older demographic in homes”* (M, 67) and *“people in hospital”* (F, 65) as among those needing an increased protein intake, but the majority did not believe that older adults needed additional protein. This was echoed by older focus group participants stating that requirements “*probably stays the same until you are 60/70 and then you probably need less of everything*” (M, 69). A lack of awareness about the increased requirements for protein associated with ageing has been previously reported (23, 31) and highlights the need for education for this demographic.

Both age groups demonstrated a poor understanding of protein functions, only associating protein with muscle strength and struggling to name any further functions of this macronutrient. Some participants may have linked this to building muscle or bulking, rather than maintaining muscle with age; it is possible that this is one of the causes for the reluctance to increasing protein intake (discussed in Section 4.3), as individuals are not motivated to increase their protein intake if they are not aware of its functions and potential health benefits. This was previously identified as a barrier to protein consumption by Buhl et al. (34); the authors noted that increasing awareness and knowledge about nutrition recommendations may help address misperceptions of healthy diet in ageing and empower older adults to make behaviour changes (34). It is possible that this low knowledge is specific to the UK as studies in other countries have demonstrated better levels of awareness of protein functions (35). Food knowledge has been shown to have an important role in shaping dietary behaviour (19) and was identified as having the potential to influence openness and motivation to include high protein ingredients in meals (23). In light of this, interventions could focus on increasing knowledge, as a means to increase consumers’ motivation to independently increase their protein intake (32).

### 4.2 Awareness of protein sources

Both the survey and focus groups indicated that participants believed that animal products were better sources of protein compared with plant-based options. In the survey, participants stated that meat, fish, dairy products, and eggs were a good source of protein, whilst plant-based sources were less frequently selected. This is in keeping with previous literature (22). In the focus groups, younger adults also mentioned meat analogues as a good source of protein; this was not mentioned in the older adult sessions. Other than meat analogues, in both age-groups, individuals only cited plant-based protein sources if they were or had been vegetarian/ vegan. Vegetarians had more awareness of nuts, seeds, legumes and beans as protein sources. This low populational knowledge a potential barrier to plant-based protein consumption if individuals do not know what foods fall into this category, or if they are only thinking of meat analogues as these often have a higher cost (19). Plant-based diets have previously been identified as a gap in evidence-based interventions for public behavioural change (36). Cost was identified as a barrier to high protein consumption throughout the study with 16 references to cost in focus groups. This is supported by previous literature, as older adults have a lower average weekly food expenditure compared with younger adults (23, 37). The authors recognise that this barrier also prevents the consumption of healthy foods and other nutrients across the diet (38). Similarly, the observation of a willingness to consume more healthy options being limited by a lack of knowledge of food sources has been reported previously with regards to healthy eating (39). This highlights the importance of nutritional education and that this phenomenon is not unique to protein and is likely to be limiting healthy intake across all aspects of the diet.

The current study did not ask about the number of protein sources consumed on a typical day. However, previous studies have reported that older adults consume most of their daily protein in one meal (9) and consider this to be sufficient for achieving their daily protein needs (32, 40). This emphasises the increased risk of protein deficiency within this demographic and the need for education. The importance of ease, low effort, and minimal waste was also been emphasised as driving factors for acceptance of high protein foods by older adults (41): it is possible that these factors for acceptance may vary within older adults during advanced ageing. This supports the suggestion of sub-dividing older adult groups for future research to understand key factors for younger-old compared with older-old.

### 4.3 Attitudes towards supplementation and fortification

Supplementation showed a higher prevalence among younger adults in the focus groups. Justifications for these choices includes convenience (especially for protein bars and ready-made shakes), control (for self-made protein shakes), and the ability to bulk buy products. Older adults did not report consuming protein supplements. The biggest barrier to consumption identified by older adults was not believing they required supplementation if they were eating a balanced diet. The belief that supplementation is not needed in a healthy diet has been cited previously (13, 25, 31). As discussed in Section 4.1, this lack of motivation may reflect low levels of understanding about protein requirements and functions. Previous research into older adults reported that 67.4% participants considered the amount of protein in their diet to be “just about right,” despite being unaware of protein requirements or functions (32). An additional barrier against protein supplementation within the older age group is their association with frailty and chronic illness. A few older adults acknowledged that protein supplements might be useful for “*the older demographic in homes*” (M, 67) and “*people in hospital*” (F, 65). However, they did not classify themselves as among those needing supplementation as this was an effect of frailty rather than chronological age. This association between supplements and end-of-life care has been previously reported (42) and is known to prevent uptake by participants who regard themselves as healthy and do not want to be associated with products for end-of-life. This is a significant barrier preventing the consumption of high-protein foods by older adults and is a relevant area for educational intervention to elicit change. Future research could be conducted on older-old adults (85+) to understand how these attitudes change with advanced ageing, as well as those in supported living facilities who are more likely to experience the frailty discussed.

Older adults also expressed concerns about fortified foods, which has been cited previously (35). This was based upon the inclusion of unknown ingredients and their potential impact on health with one individual saying “*we should be eating food that is natural, not full of bits have been added by somebody in a production unit somewhere*” (F, 76). The detrimental effects of ultra-processed foods on health was raised as an issue in both focus groups with older adults and was unanimously agreed to be of concern to these participants: this is in agreement with previous literature (31, 35). Lack of trust and scepticism towards functional foods due to a belief they are high in processing, additives, and preservatives was reported in focus groups with Irish older adults (22), as well as Dutch participants citing scepticism of a product’s claims (19, 25). Other concerns about fortified foods included a fear that they could “*overdose*” (F, 66). This was echoed by young adults: “*is there, like, too much protein you can have in your diet?*” (M,25). Survey participants also indicated that they wanted to learn more about if there are any issues with consuming excess protein. These attitudes highlight a need for educational messaging to alleviate concerns about excess consumption and encourage intake of high-protein foods.

In focus groups, participants were asked to give a preference between supplements and fortification. This was one of the most age-polarised topics seen in the focus groups: younger adults preferred supplements such as protein shakes and bars, citing convenience, control and the ability to bulk buy as factors motivating their decision; older adults preferred fortification as this requires a change in purchasing rather than in eating habits. This preference towards familiar whole-foods agrees with the literature (13, 25, 43). In the current study, the example of switching to a high-protein bread was given as an easy switch to increase protein intake: this had a positive reception with the older adults. However, concerns about sensory impacts of the increased protein were reported as important in the acceptance of fortified foods (22, 44). Taste and texture of high protein foods is known to be important in their acceptance and adoption into dietary patterns, especially for older adults (13, 31, 41, 45). This suggests that the main barriers to increasing protein intake differ with age, with younger adults being limited by time and convenience, whereas older adults are limited by established dietary habits and an unwillingness to change. The ingrained nature of habits and routines was particularly apparent in the focus groups of older adults: “*you know [at] our age, certainly for myself, it’s proven much more my habit than anything else*” (M, 69) and “*I think the only thing is that habit and convenience are often much bigger factors than what is good for you”* (F, 66). Habit and nostalgia have been cited previously as a key reason for non-consumption of high-protein foods (44). Confusion regarding the intended time of consumption of protein supplements around current eating patterns has also been cited as a barrier to their introduction (31): previous research showed that participants most favourably considered a high-protein product in the snacking category (35), meaning this may be the easiest avenue for intervention as opposed to changing meals. This reduction in willingness to change dietary behaviours with increasing age has been reported previously (24, 32), as well as a preference for foods that are already regularly consumed as a carrier to protein enrichment (13, 25). Food familiarity was highlighted as a key facilitator for the uptake of fortified foods (23, 44).

### 4.4 The effectiveness of educational materials to increase intake

When discussing the educational materials provided in our study, both age-groups indicated a preference for the infographic out of the two but when placed in the wider context of resources an age-associated difference was seen. The older adults articulated a preference for paper leaflets, citing the ability to “*re-read things [and] go back to things that kind of caught my interest*” (M, 68). This intention to revisit information may be reflective of a greater leisure time availability and a reduced exposure to information and content, compared with young adults who described the plethora of online dietary information as “*overwhelming*” (F, 25). Previous research also showed a preference for written information by older adults, with one study identifying newspapers as a familiar communication tool for 35 out of 40 older adults (24). As a consequence of this higher content consumption, younger adults expressed a preference for concise and easily digestible information formats: the majority favoured online resources such as short videos accompanied by links to further information or short amounts of text. The amount of information available was identified as a potential barrier to behavioural change due to the presence of conflicting messages and general confusion. When discussing the use of social media, younger adults mentioned the issues associated with influencers often having no nutritional qualifications and being financially motivated to promote a product, but also that they felt this was a good source of information in digestible and clear formats. Overall, they suggested a mistrust of platforms such as Instagram and influencers, rather than receiving educational information in a video format. More research is needed to fully understand how social media platforms can be harnessed for nutritional education. It is known that role models have a high potential to modify dietary behaviours (19), so it is possible that this presents an opportunity for social media interventions after trust has been built. It is also recognised that social media has created a growing trend of health conscious active young people through a fitness-focused culture, that should be explore further in future studies. This highlights the need for age-specific educational materials as the formats that will lead to maximum engagement are different for each age group; the need for context-specific nutritional education tools has been suggested previously to support healthy eating (39). It also highlights a difference in time availability meaning resources targeted for older age groups can go into more detail as users are likely to revisit it, whereas information for younger adults needs to be received within the first viewing.

From the survey findings, it was evident that participants trusted information from scientists or research institutions and evidence-based organisations. From the focus groups, both age groups raised trust as an important consideration in the use of educational materials. Both age groups concurred that they trusted the information leaflet given due to the presence of an affiliation on the documents. However, the context for this information was important: participants trusted the university where the leaflets were distributed and said that if given the same material outside a supermarket, they would be more sceptical for possible brand sponsorship or product placement. This highlights the importance of context in providing educational materials to ensure that individuals engage with the message. It is also important for educational materials to address a gap in current materials. The trust given to both health professionals and governing bodies mean these groups may be best placed to offer educational input. However, it was previously noted that healthcare professionals also require education of nutritional needs of older adults in order to implement educational strategies effectively (5). Due to the higher prevalence of chronic conditions that may impact dietary choices for older adults, it would also be beneficial for these educational strategies to contain information on replacement foods that are cut out for medical reasons (44).

In terms of what information they would like to know about protein, participants expressed their desire to learn more about plant-based protein sources in the survey. It is possible that this was affected by the survey demographics, but the authors still consider this finding relevant especially for females which from 74.8% of the survey participants. In the focus groups, younger adults listed this knowledge gap as a barrier to increasing their protein consumption: “*not knowing what the other proteins are … other than meat*” (F, 24). There is a deficiency in knowledge regarding non-animal protein sources, presenting an opportunity for public education (15, 19, 20). Recipes, meal examples, and product lists have previously been recommended as methods to address a lack of knowledge of high protein foods (19, 23), but research is limited on their efficacy for inciting long-term change. Whilst discussions indicated that increased knowledge was likely to affect dietary habits and increase protein intake, the current study did not measure behavioural change. This is significant as research often reports an intention-behaviour gap where real differences are less than those predicted from intentions alone (39, 46). Longitudinal studies would be required to fully understand the practical effect of education interventions on dietary habits.

### 4.5 Prevalence of confusion within the general public

A common theme of confusion was observed across all focus groups with many participants prefacing their statements with phrases such as “*I do not know but*” (F, 75) or “*I’m not sure*” (F, 76), or concluding their statements with admissions of uncertainty such as “*that could be my lack of knowledge*” (F, 25) or “*but I do not know the facts*” (F, 25). Older adults mentioned that much of their confusion has stemmed from differing nutritional information being displayed on various news outlets. It was noted that changing guidelines over time had led to a general mistrust in information being promoted by researchers and governing bodies: “*This is good for you. And then a year later, this is going to kill you*” (F, 75). This distrust of the modern food industry directly echoes the findings of previous studies (31) including Linschooten et al. (24) with a participant stating “*Three years ago, eggs were considered to be bad, and now you can have 3 eggs a day without health consequences*.” It was previously argued that older adults were more greatly affected by changing health information due to increased exposure to contrasting messaging over time (44). This generational distrust has led to a lack of motivation to engage with educational materials and limits the ability of these interventions to incite behavioural change. This was previously reported as an apathetic response to scientific evidence on nutritional issues due to the rapidly changing nature of recommendations (47). This highlights the importance of clear messaging to overcome this barrier for older adults as they have a general distrust of nutritional advice. The issue of low participant trust in product claims has been discussed previously, with many high profile cases such as the horsemeat scandal being cited as reducing trust in the food industry (25).

Younger adults also cited confusion, but this was attributed more to a lack of nutritional learning at school. This was exacerbated by the volume of conflicting content on social media. The confusion and low levels of knowledge seen across both age groups highlights the need for educational intervention to increase awareness and consumption of dietary protein. However, the barriers to these changes are different between the age groups, driving a need for age-group specific marketing and messaging to lead to higher levels of engagement at a population level.

### 4.6 The knowledge-confidence gap present in older adults

In the current study an age-associated difference was observed regarding confidence in nutritional knowledge. Whilst both age groups admitted to feelings of confusion or uncertainty at times (see Section 4.5), older adults were more assured and confident in their knowledge. As a result, they explained they would only trust educational materials that gave advice they already knew. This may be a result of the lack of trust in public messaging leading many older adults to rely on their own perceptions of healthy eating rather than following expert advice: older adults are greatly affected by changing health information due to increased exposure to contrasting messaging over time (44). This was highlighted by the participants’ lack of motivation to engage with the educational materials and a lower desire to increase their knowledge about protein: “*I think if it said something that was surprising, then I would look, you know who wrote it*?” (F, 70). This suggests that they would not trust new information that contradicts their existing beliefs; this is a barrier to educating this demographic as new information will be met with scepticism and distrust, which is likely to impact engagement. This was supported by Linschooten et al. (24) which reported that older adults believed that they already had sufficient nutrition knowledge to know what they need, and had a generational mistrust of health claims on products due to past food safety scandals (24, 44). This mistrust of nutritional information presents a significant barrier for this demographic and should be acknowledged in the design of future interventions through consistent, clear messaging. Future studies with older adults should be established to further understand this knowledge-confidence gap and better understand the correct interventional tools to address it.

### 4.7 Study limitations and future directions

In the focus groups, younger adults were classified as 18–30 and older adults were classified as 65+. This classification was chosen to increase compatibility with existing work in the literature; however, this low age cut-off for older adults has the potential to span over 30 years, meaning this is a highly heterogenous group. It is possible that individuals within this group will vary in both their preferences and attitudes as a result of the wide age range. Future research should address this by sub-dividing the group of older adults to further understand the attitudes of this demographic, specifically for younger-old and older-old. Through this a more complete image of older adult preferences could be obtained. Additionally, our research did not investigate the attitudes of middle-aged adults or at what age attitudes begin to change: the age group 55–65 were identified by Jamshidvand et al. (22) as an important research demographic as the “future old” group. This group may be more open to behavioural change compared with older adults, presenting a novel opportunity for education intervention, which should be investigated through focus groups in future research. It is also possible that patient groups may demonstrate increased receptivity towards diet change. Previous research shows that product choices of consumers who experience health problems are more likely to be driven by health-related considerations (48). It is possible that research into older adult patient groups may lead to better engagement with education interventions and uptake.

Whilst the survey was open to all adults living in the UK, the authors recognise that the majority of respondents were located in the Southeast of England (66.3%). Therefore, it is possible that this data is only representative of this area, and thus transferring conclusions to the wider UK landscape should be made with caution. Moreover, the survey participants were predominantly women (74.8%) and younger adults which limits the representativeness of the survey findings across the demographic groups. Whilst we acknowledge that the findings may not fully capture variation across demographic groups, the analysis focused on the overall patterns providing a more robust account of the responses. The potential effect of the imbalance in the survey distribution has been taken into account when interpreting responses. In addition, we ensured a balanced number of participants in terms of age and gender for the subsequent focus groups stage allowing these demographic differences to be seen. Another limitation of the survey design was that participants were required to answer each question, meaning they may have guessed answers: it is not possible to ascertain the levels of certainty in participant responses. It is also likely that levels of public knowledge are lower than those indicated in the focus groups, as participants who signed up for a session on protein are likely to have higher interest and engagement in nutrition than the general public. Finally, the survey was conducted online to enable anyone across the UK to participate. However, this may have prevented those with reduced digital literacy to take part, meaning future surveys should be conducted in a hybrid format to ensure these opinions are also represented.

Finally, the authors asked focus group participants if they planned to change their dietary habits based on the educational material they had been given. However, it was outside the scope of the study to record any dietary changes: future research could further investigate the effects of education through longitudinal studies including food diaries before and after the intervention. In our study it is possible that participants said they intended to change their dietary habits but that this did not result in a change in eating behaviour; a pattern known as the intention-behaviour gap (46). This gap is a cross-cultural phenomenon, as highlighted through studies into healthy eating in Morocco (39). It would also be useful to understand how current eating habits may have affected their views on protein, which cannot be commented on in the present study: this could be addressed in future work.

## 5 Conclusion

The present highlights a knowledge deficit independent of age for protein requirements, functions and sources. Levels of knowledge for all topics were low with focus group participants demonstrating a poor understanding of the physiological uses of protein. The authors suggest that the low levels of understanding of the health benefits of protein are detrimentally impacting the engagement with educational messaging and content desired to elicit behavioural change. Whilst our study identified a widespread knowledge gap, older adults were more confident in their nutritional knowledge and as a result, were more resistant to new information. Trust of the food system and nutritional recommendations was also identified as a significant barrier to eliciting behavioural change. Other barriers identified were fears about overdosing on protein, ultra-processed foods, unknown ingredients and additives: this was particularly seen with regards to protein supplementation and fortification, where levels of consumption are lower in the older demographic. The present study highlighted the effect of age on preferences for these materials. Younger adults were more likely to prefer information in a video format, with the option of additional supplementary text. By contrast, older adults prefer a text-based resource, giving them the ability to return to the same resource multiple times to gain its full meaning.

This study has highlighted the need for education to increase the public’s knowledge of protein sources and functions. It has also discussed the age-associated preferences for educational materials and the potential positive use of technology to increase engagement. This knowledge will facilitate the development of better educational materials and subsequently increase the dietary knowledge of UK consumers.

## Data Availability

The raw data supporting the conclusions of this article will be made available by the authors, without undue reservation.
