# Supplementary material for: “Why would I need more?”: How age shapes UK consumers’ perceptions of dietary protein and responses to educational interventions
Source: Front Nutr. 2026 Jul 17;13:1893639. doi: 10.3389/fnut.2026.1893639 (PMC13426273; doi:10.3389/fnut.2026.1893639)
Supplement: Supplementary file 1 [file Table_1.DOCX]

Supplementary Material

**Table S1.** Questions and answer options included in online survey with UK adults to understand knowledge of protein and attitudes to educational interventions to increase protein intake (demographic questions not included).

| **Question** | **Answer options** |
| --- | --- |
| Which of the following do you think are good sources of protein? Please check all that apply. | Meat, fish, dairy products, eggs, beans and pulses, nuts, tofu, broccoli, edamame beans, pasta, potatoes, salads, lemons and oranges |
| What do you think the best source of protein is? | (open text response) |
| Do you think dietary sources are sufficient for protein requirements? | Yes, no, unsure |
| What do you think the daily protein recommended intake is for adults? Please select one response. | 0.5g per kg body weight, 0.75g per kg body weight, 1g per kg body weight, 1.25g per kg body weight, I don’t know |
| Do you think protein requirements change with age? | Yes, no, unsure |
| If yes, do you think adults over 65 require more or less protein than younger adults (20-30 years old)? Please select one response. | More, less, the same |
| Which population group do you think are most at risk of protein deficiency? Please choose one response. | Children, adults over 65, pregnant and lactating women, not sure |
| Do you currently take protein supplements? | Yes, no, unsure |
| If yes, why do you currently take protein supplements? | Recommended by doctor, general health and wellbeing, don’t think I get enough in my diet, energy, weight gain, immune function, sports performance, memory improvement, other |
| What type of protein supplements do you take? | Whey protein, casein protein, plant-based protein (eg. Soya, pea) |
| Do you think there is any harm in taking protein supplements? | Yes, no, unsure |
| Do you know what fortified foods are? | Yes, no, unsure |
| Protein fortified foods are products that have additional high protein ingredients in order to increase protein without increasing the portion size. Do you normally purchase and consume fortified products? | Yes, no, unsure |
| If no, what obstacles prevent you from trying fortified foods? | Highly processed, expensive, taste is poor, not healthy, unnecessary, other |
| What do you think are the health benefits of protein? | Muscle strength, immune function, injury recovery, decreased appetite, stronger nails, reduced cholesterol, increased metabolism and fat burning, increased memory and brain function, shiny hair, reduced blood pressure, improved sleep |
| Which of the above factors would motivate you the most to consume more protein? | Muscle strength, immune function, injury recovery, decreased appetite, stronger nails, reduced cholesterol, increased metabolism and fat burning, increased memory and brain function, shiny hair, reduced blood pressure, improved sleep |
| Would you like to learn more about the benefits of protein for health and where to find it in your diet? | Yes, no, unsure |
| What other information would you like to know regarding protein? | (open text response) |
| Thinking about different sources of information available, which do you trust more? Please check all that apply. | Government output, evidence-based organisations, journalists, friends and family, scientists or research institutions, independent consumer organisations, social media influencers, none, other |
| If you were to receive information about protein consumption what would be your preferred format? Please rank this from your favourite (1) to least favourite (5) option. | Paper leaflet, online information sheet, video to watch online, face to face group session, face to face individual session |


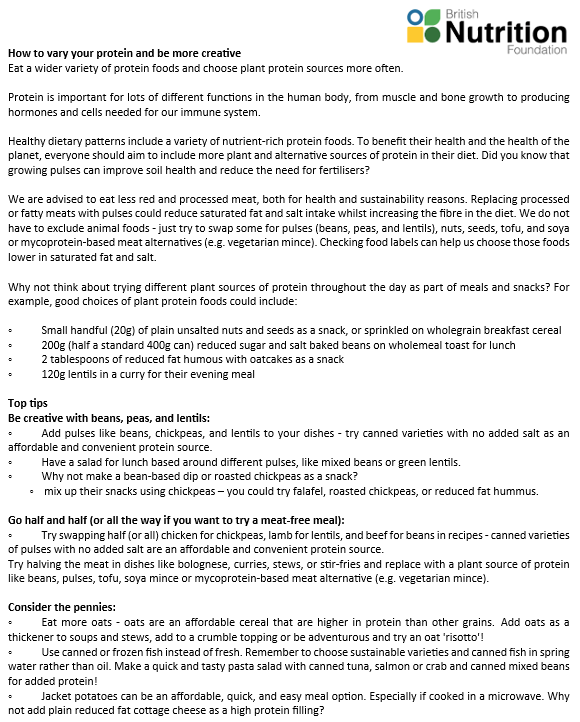


**Figure S1.** Information adapted from British Nutrition Foundation:

<https://www.nutrition.org.uk/media/a5cfam2v/hew-secondary-guide-23.pdf> -


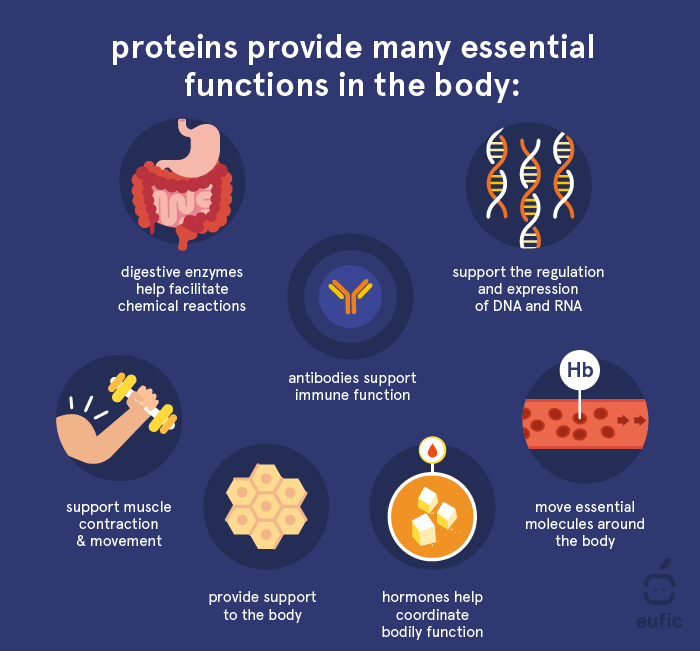

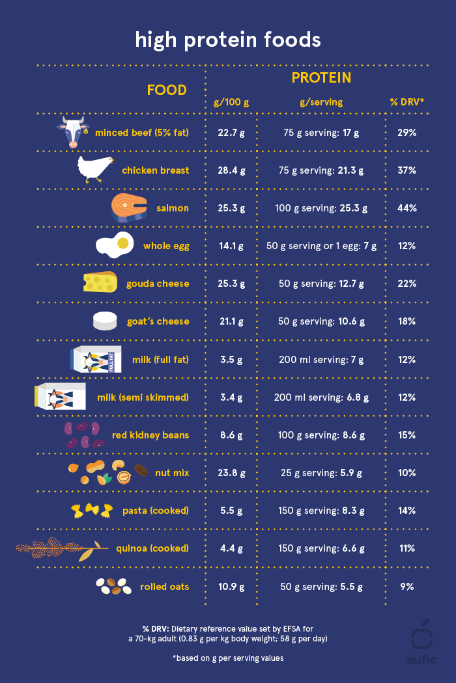


**What are the health benefits of protein?**

Eating enough protein to meet our bodies requirements is important for many body functions. However, there is evidence to suggest that in certain situations increasing protein intake above required levels could provide additional health benefits.

**Protein and sarcopenia**

- Sarcopenia is a disorder characterised by the progressive loss of muscle mass and physical function that is commonly associated with older adults.
- Sarcopenia is associated with increased frailty, risk of falls, functional decline and even early death.
- As protein is essential for the repair and maintenance of muscle mass, it is no surprise that low intake of protein is associated with an increased risk of developing sarcopenia.[https://www.eufic.org/en/whats-in-food/article/what-are-proteins-and-what-is-their-function-in-the-body](https://www.eufic.org/en/whats-in-food/article/what-are-proteins-and-what-is-their-function-in-the-body#ref6)
- Similarly, increasing protein intake, as well as increasing [physical activity](https://www.eufic.org/en/healthy-living/article/physical-activity-and-health) can help maintain muscle mass and strength as we age, decreasing our risk of sarcopenia and [skeletal disorders](https://www.eufic.org/en/healthy-living/article/healthy-bones-for-life).

**Protein and athletic performance**

- Protein plays a key role in helping to repair and strengthen muscle tissue after exercise.
- Although protein is critical for building muscle, to maximise the benefits it should be considered in the context of the whole diet, which includes the right amount of carbohydrates, fats, vitamins and minerals.
- Optimum protein intake will depend on the type (e.g. endurance or resistance training), duration and intensity of exercise, with more not always being better. A protein intake of 1.4–2.0 g per kg body weight a day (e.g. 98 – 140 g per day for a 70 kg adult) is thought to be sufficient to meet the needs for most exercising individuals.

**Figure S2.** Information leaflet adapted from the European Food Information Council <https://www.eufic.org/en/whats-in-food/article/what-are-proteins-and-what-is-their-function-in-the-body>
